# Kolmogorov–Arnold Network in the Fault Diagnosis of Oil-Immersed Power Transformers

**DOI:** 10.3390/s24237585

**Published:** 2024-11-27

**Authors:** Thales W. Cabral, Felippe V. Gomes, Eduardo R. de Lima, José C. S. S. Filho, Luís G. P. Meloni

**Affiliations:** 1 Department of Communications, School of Electrical and Computer Engineering, University of Campinas, Campinas 13083-852, Brazil; t264377@dac.unicamp.br (T.W.C.); jcssf@unicamp.br (J.C.S.S.F.); 2 Transmissora Aliança de Energia Elétrica S.A.—TAESA, Praça Quinze de Novembro, Centro, Rio de Janeiro 20010-010, Brazil; felippe.gomes@taesa.com.br; 3 Department of Hardware Design, Instituto de Pesquisa Eldorado, Campinas 13083-898, Brazil; eduardo.lima@eldorado.org.br

**Keywords:** Kolmogorov–Arnold Network, power transformers, DGA sensoring, fault diagnosis, artificial intelligence

## Abstract

Instabilities in energy supply caused by equipment failures, particularly in power transformers, can significantly impact efficiency and lead to shutdowns, which can affect the population. To address this, researchers have developed fault diagnosis strategies for oil-immersed power transformers using dissolved gas analysis (DGA) to enhance reliability and environmental responsibility. However, the fault diagnosis of oil-immersed power transformers has not been exhaustively investigated. There are gaps related to real scenarios with imbalanced datasets, such as the reliability and robustness of fault diagnosis modules. Strategies with more robust models increase the overall performance of the entire system. To address this issue, we propose a novel approach based on Kolmogorov–Arnold Network (KAN) for the fault diagnosis of power transformers. Our work is the first to employ a dedicated KAN in an imbalanced data real-world scenario, named KAN_Diag_, while also applying the synthetic minority based on probabilistic distribution (SyMProD) technique for balancing the data in the fault diagnosis. Our findings reveal that this pioneering employment of KAN_Diag_ achieved the minimal value of Hamming loss—0.0323—which minimized the classification error, guaranteeing enhanced reliability for the whole system. This ground-breaking implementation of KAN_Diag_ achieved the highest value of weighted average F_1_-Score—96.8455%—ensuring the solidity of the approach in the real imbalanced data scenario. In addition, KAN_Diag_ gave the highest value for accuracy—96.7728%—demonstrating the robustness of the entire system. Some key outcomes revealed gains of 68.61 percentage points for KAN_Diag_ in the fault diagnosis. These advancements emphasize the efficiency and robustness of the proposed system.

## 1. Introduction

The instability of energy supply caused by malfunctioning equipment in electrical substations has driven energy companies to strive for a more reliable and sustainable electricity supply. Failures in critical components, such as power transformers, can compromise the efficiency of the electrical network or even lead to its failure [1]. To prevent such situations, electric power companies are motivated to invest in monitoring systems and data processing technologies, with an emphasis on developing solutions for power transformer status diagnosis [2].

Across the years, the scientific community has developed strategies for monitoring, diagnosing faults, and insulation degradation in power transformers. However, according to [3], the dissolved gas analysis (DGA) approach is one of the most commonly employed techniques for analyzing transformer oil in order to identify early-stage faults in industrial practice. The primary concept behind DGA is to assess the concentrations of different gases dissolved in transformer oil. These gases are produced due to events such as partial discharges, overheating, and electric arcing. The types and concentrations of gases dissolved in transformer oil provide valuable insights into potential faults. This information allows for proactive maintenance and repair strategies, helping to prevent serious damage and extend the transformer’s operational lifespan.

DGA relies on the concentration of certain gases—known as key gases—to assess the condition of a transformer. These key gases include ethylene (C_2_H_4_), methane (CH_4_), ethane (C_2_H_6_), acetylene (C_2_H_2_), hydrogen (H_2_), carbon monoxide (CO), and carbon dioxide (CO_2_). From these gas concentrations, the IEC 60599 standard [4] provides a framework for classifying faults in equipment based on inspection results. The IEC 60599 standard details how the concentrations of dissolved gases can be analyzed to evaluate the fault condition of equipment. These indicators enable the determination of the health condition of a transformer. Fault diagnosis assessment provides a holistic analysis of the overall condition of a power transformer.

The initial DGA approaches to fault diagnosis are known as interpretation methods [5]. Duval, for example, proposed a geometric representation in the form of a triangle with internal regions and their boundaries, where these regions represent categories of faults [6]. This approach only classifies faults and is unable to detect the normal state. In contrast, there are methods in the literature able to classify the normal state, such as the ASTM standard [7], IEC standard [8], CEGB standard [7], and Rogers ratio [9]. However, as per [10], these methods have certain limitations, such as uncoded ratios and varying thresholds, which can result in incorrect and inconsistent fault diagnoses. The state-of-the-art methods now integrate AI models into their solutions for fault diagnosis [11]. Currently, the most modern works employ decision tree (DT) [12], random forest (RF) [13], *k*-nearest neighbor (*k*-NN) [14], support vector machine (SVM) [11,15], extreme learning machine (ELM) [16], artificial neural network (ANN) [17,18], and convolutional neural network (CNN) [3] methods. Here, the challenge lies in applying a robust model that makes the adopted method more intelligent and suitable for the application.

This work proposes a comprehensive approach to assessing the fault diagnosis of power transformers immersed in oil. The proposed system comprises a module exclusively for fault diagnosis in the power transformer.

Our system is the first to employ a Kolmogorov–Arnold Network (KAN) for classification in a real-world scenario under an imbalanced dataset in the fault diagnosis module (KAN_Diag_).

The proposed system is under development as part of the research project entitled “Artificial Intelligence Techniques Applied to Predictive Fault Analysis in Electric Power Substations”. This initiative entails cooperation between the University of Campinas (UNICAMP), the Instituto de Pesquisas Eldorado, and Transmissora Aliança de Energia Elétrica S.A. (TAESA).

### Major Contributions

The key contributions of our work are as follows:

1. **Novel approach for the fault diagnosis of oil-immersed power transformers:** Our work proposes a pioneering system for the fault diagnosis of oil-immersed power transformers based on the KAN. The proposed system comprises a exclusive module for fault diagnosis in the power transformer. The system is the first to employ a KAN for classification of an imbalanced dataset in the fault diagnosis module (KAN_Diag_). Due to this original proposal, this paper also introduces a pioneering analysis of the KAN_Diag_ architecture applied to a dataset collected under realistic conditions of imbalance from power transformers monitored by the TAESA company.
2. **Practical implications:** Key results revealed that KAN_Diag_ reduced the classification error by 17.53 pps in Hamming loss compared to the ASTM standard, improved F1 by 20.70 pps, and surpassed the ASTM standard by 17.53 pps in accuracy. KAN_Diag_ consistently outperformed other interpretation methods, such as the IEC standard, CEGB standard, and Rogers ratio. Compared to machine learning models, KAN_Diag_ reduced the classification error by 3.09 pps relative to ELM, improved F_1_ by 2.96 pps, and exceeded ELM by 3.10 pps in accuracy. It also outperformed the other ML models in this study, including DT and *k*-NN. Notably, KAN_Diag_ showed a 10.01 pps advantage over a direct rival, the CNN from reference [3]. In addition, the pioneering system achieved a minor Hamming loss, and the highest F_1_ and accuracy for the fault diagnosis module—0.0323, 96.8455%, and 96.7728%, respectively.In practice, the proposed approach is more suitable for implementation in the field and maintains high performance, as highlighted by the results. These practical implications stem from the advanced system and the adoption of more robust and reliable models, resulting in notable enhancements in the performance of the fault diagnosis of oil-immersed power transformers and showcasing the effectiveness and refinement of the proposed approach.
3. **Advances in the fault diagnosis of oil-immersed power transformers field:** Our study makes significant contributions to key aspects of the fault diagnosis for oil-immersed power transformers, including data preprocessing, machine learning architectures, levels of robustness, and a reduced implementation complexity. Additionally, it addresses existing gaps in current methodologies by enhancing equipment fault detection through KAN_Diag_, thereby boosting the overall performance of the system with more robust models. Notably, the proposed approach sets a new benchmark for ML architectures in the fault diagnosis of oil-immersed power transformers. This advancement can promote the development of more robust, accurate, and reliable systems, having a positive impact on both academic research and, especially, practical applications in the power supply industry.
4. **A survey of modern approaches:** We present a accurate bibliographic review of current methods, focusing on essential aspects such as the implementation of the fault diagnosis module, input variables for this module, oversampling techniques, the decision maker, and system robustness. This survey sheds light on recent developments in fault diagnosis technologies, emphasizing the critical elements that influence the performance and practicality of such systems.

The organization of the remaining sections is as follows: Section 2 provides a comprehensive background to set the context for this study. Section 3 provides a detailed description of the proposed system, including its processing flow, data balancing technique, and the implemented models. Section 4 outlines the collected dataset, the metrics used in this study, and their rationale; discusses the results obtained with the proposed method; and provides an analysis of the findings, along with new insights. Section 5 summarizes the conclusions of the manuscript, presents the implications of the proposed strategy, highlights key findings, and identifies promising aspects of the proposed system.

## 2. Theoretical Background

This section offers a comprehensive review of current methods for fault diagnosis in oil-immersed power transformers and presents key concepts in the field.

### 2.1. Literature Survey

- **Fault diagnosis module:** The fault diagnosis module is an essential component in DGA-based solutions, because of its critical role in transformer maintenance. This module aims to detect anomalies in equipment performance by using dissolved gas analysis (DGA) to identify internal faults. Such a module relies on DGA, considering parameters that reflect the internal condition of the equipment, i.e., gas concentrations. However, no consensus exists on how many gases researchers must use as inputs to a module [19,20,21,22,23]. In the work [24], for example, the authors explored the relationship between oil quality and its impact on dissolved gases, examining how these factors influence them. In their approach, oil’s physicochemical properties—such as acidity, breakdown voltage, water content, interfacial tension, density, and oil power factor—are used as inputs for an MLP, where the estimation of dissolved gas concentrations is the network’s output. Furthermore, traditional methods, including key gas, Dörnenburg, and Rogers ratio techniques, occasionally fall into an “indecision zone”, where they cannot provide a definitive diagnosis. This limitation in traditional methods may lead to undetected or misclassified incipient faults, ultimately impacting the effectiveness of fault diagnostics in power transformers. It is relevant to highlight that, in most studies, the selected gas concentrations undergo data processing before inputting into the module. There is also no consensus on the best ML architecture for decision-making [3,25,26,27,28]. Indeed, state-of-the-art methods typically employ AI models. This module enables early problem detection, which is essential for preventing catastrophic transformer failures, thereby enhancing the safety and longevity of the equipment.
- **Input variable for fault diagnosis module:** The input variables for fault diagnosis modules play a crucial role in the accuracy of the analyses. The combination of gas concentrations can indicate the presence of faults, providing reliable diagnostics for preventive maintenance. According to Table 1, studies [19,23,25,26,27,28] have focused on the use of five gases (C_2_H_4_, CH_4_, C_2_H_6_, C_2_H_2_, H_2_). However, variations in gas combinations may exist, such as in [21], which employed (C_2_H_4_, CH_4_, C_2_H_6_, H_2_, CO). Different gas combinations can detect various types of faults. For example, hydrogen (H_2_) and methane (CH_4_) are associated with electrical discharges, while ethylene (C_2_H_4_) and acetylene (C_2_H_2_) indicate high-intensity arc faults. Even for binary classification (normal or fault), which is the focus of this manuscript, employing the seven gases—C_2_H_4_, CH_4_, C_2_H_6_, C_2_H_2_, H_2_, CO, CO_2_—is advantageous. According to [3], utilizing the current seven key gases is typically documented as an effective approach for fault detection. For instance, the work in [20] employed all seven gases, enhancing the fault detection capability by providing more data to the model as more gases were considered. The combination of different gases demonstrates that, the more variables are included in the system, the greater the predictive capacity of the diagnostic model, thus increasing the system’s accuracy and reliability. In particular, the work in [20] emphasized that using seven gases instead of just five can significantly improve diagnostic accuracy, as it offers a more comprehensive view of the degradation processes occurring within the transformer;
- **Oversampler:** Data imbalance presents a frequent challenge in real-world fault diagnosis applications, because fault and malfunction occurrences are rarer than normal working. Researchers often apply oversampling techniques to address this issue [3,26]. These techniques generate synthetic samples for the minority class (faults), helping to balance the dataset and improve the performance of learning algorithms within the fault diagnosis module. By employing such techniques, researchers can reduce the bias in predictive models and enhance overall performance, especially in scenarios with limited and imbalanced data. As listed in Table 1, only the studies in [3,26] and the present work implemented oversampling techniques. Reference [26] tested the oversampling techniques SMOTE, ADASYN, GAN, and DCGAN. The study [3] employed SMOTE in the fault diagnosis module. Our proposed approach uses SyMProD to balance data within the fault diagnosis module;
- **Decision maker:** The decision-making mechanisms used in the literature vary widely across approaches. Some studies have employed interpretation approaches, such as the ASTM standard [7], IEC standard [8], CEGB standard [7], or Rogers ratio [9]. Other studies used modified IECM and modified Rogers ratio [19], and modified triangle diagnosis [23]. In contrast, other studies employed techniques derived from fuzzy logic, such as ANFIS in [20] and fuzzy C-means in [25]. However, state-of-the-art studies incorporated ML models into their frameworks. In [21], the authors employed a diverse set of decision-making techniques, including DT, SVM, *k*-NN, and ensemble methods. The study in [22] explored an even broader range of decision-making tools, such as AutoWeka, naive Bayes, MLP, and SVM. Reference [26] implemented *k*-NN, SVM, and random forest (RF) models. In [27], the authors employed neural pattern recognition. In [28], the researchers applied an SVM-BA. Furthermore, cutting-edge approaches integrate a specialized module for fault diagnosis, where each approach employs advanced decision-making mechanisms. In line with this, reference [3] employed a CNN as the decision maker in their fault diagnosis module. As reported in Table 1, our proposed system employs KAN_Diag_ for the fault diagnosis, resulting in superior results to other methods;
- **Robustness:** Robustness is a key parameter that differentiates state-of-the-art methods from superior state-of-the-art methods. The studies in [3,19,20,21,22,23,25] achieved accuracy rates ranging from 28.15% to 88.90%. Consequently, they had intermediate robustness—under 90%. The studies in [26,27,28] reported accuracy rates of 90.26%, 92.80%, and 93.75%, respectively. An accuracy above 90% is impressive. Consequently, they demonstrated high robustness. Finally, our proposed system demonstrated superior robustness. Our system achieved the highest accuracy values. The proposed system showed an accuracy rate of 96.23%, surpassing the mentioned references.

### 2.2. Preprocessing

The preprocessing approach involves two key steps: first, rescaling the magnitude of the data, and second, applying normalization. As per Mirowsky and LeCunn [14], dissolved gas concentrations can exhibit asymmetric distributions, where the concentrations in ppm (parts per million) can range from low values to thousands or tens of thousands of ppm [5,29]. Because of this, they claim that the most informative characteristic of gas concentrations is their order of magnitude. However, these extreme differences between values can lead to numerical inaccuracies and overflows in learning algorithms. An effective approach to address these changes in magnitude is to apply a logarithmic transformation to rescale the data. Here, in accordance with Mirowsky and LeCunn, we employed the logarithm transformation in base 10. Thus, given a gas concentration sample *g*, this procedure transforms it into a new sample *z* using the relation $z≜\log_{10}(g)$. Additionally, to promote the stability of numerical operations in ML algorithms, thereby aiding the convergence of ML models [30], we normalized all features to have a mean of zero and unit variance throughout the entire datasetIn this second step, we apply the following normalization approach: $x≜\left(z-E\left[z\right]\right)/\sqrt{V[z]}$, where E[·] is the expectation operator and V[·] is the variance operator.

### 2.3. SyMProD Data Balancing Technique

Imbalanced datasets are common in real-world scenarios, where one class (the majority class) significantly outnumbers the other class (the minority class). Synthetic minority based on probabilistic distribution (SyMProD) is an approach designed to handle class imbalance problems in datasets. SyMProD is a oversampling technique that generates synthetic data points for the minority class to balance the dataset, creating synthetic samples by interpolating between existing minority class samples. The technique uses a probabilistic distribution to generate new samples. This probabilistic approach can provide a more diverse and representative synthetic dataset. The motivation for employing SyMProD lies in the fact that oversampling prevents overfitting [31] and provides a more balanced and diverse training set [32]. In this context, SyMProD is adaptable to different types of data and can be tailored to specific problems. This technique can generate synthetic examples of minority categories, aiding the building of more robust detection systems. Overall, SyMProD is a powerful tool for addressing class imbalances in ML and data science [32]. By leveraging probabilistic distributions, it generates synthetic data that more accurately reflect the complexity of minority class data, leading to better model performance and generalization. Where to use SyMProD? It is crucial to emphasize that, as an oversampling technique, SyMProD is applied exclusively to the training data, avoiding data leakage [33]. More details on the SyMProd algorithm can be found in [32].

### 2.4. Kolmogorov–Arnold Network (KAN)

**Figure 1:**
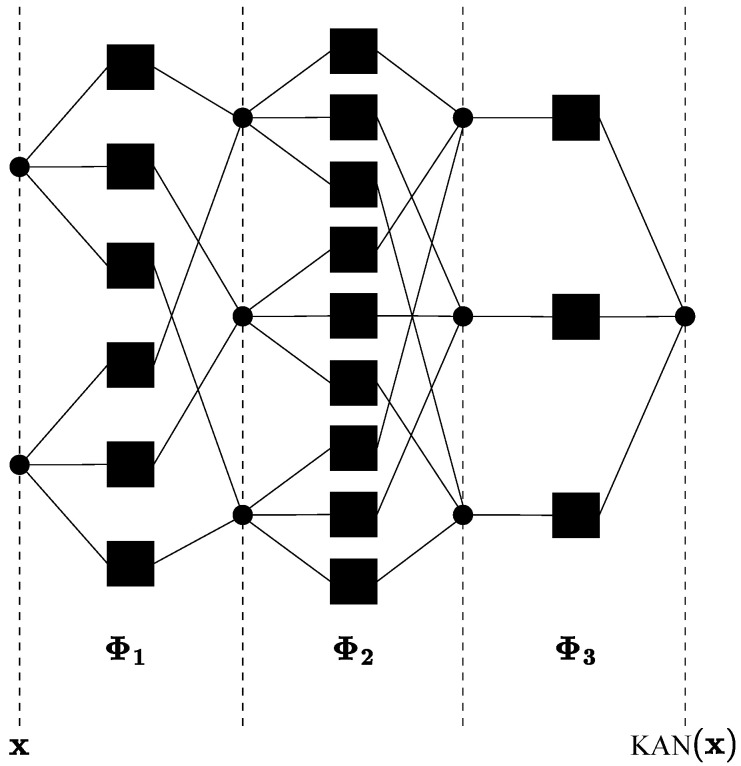
KAN architecture.

The Kolmogorov–Arnold Network (KAN) is grounded in the Kolmogorov–Arnold superposition theorem. This theorem was independently developed by the mathematicians Andrey Kolmogorov and Vladimir Arnold in the mid-20th century [34]. The Kolmogorov–Arnold theorem is a fundamental result in the theory of function approximation, stating that any continuous function of several variables can be decomposed into a sum of single-variable functions. This principle enables the construction of neural networks, known as KANs, which are capable of approximating complex functions. The theorem asserts that any multivariate function $f:{\left[0,1\right]}^{n}\to R$ can be expressed as

(1) $$f\left(x_{1},x_{2},\ldots ,x_{n}\right)=\sum_{q=1}^{2n+1}\Phi_{q}\left(\sum_{p=1}^{n}\phi_{q,p}\left(x_{p}\right)\right),$$

where continuous univariate functions exist $\phi_{q,p}:\left[0,1\right]\to R$ and $\Phi_{q}:R\to R$, called outer and inner functions, respectively. Each one-dimensional function can be represented as a B-spline curve with adjustable coefficients for the local B-spline basis functions. Splines are mathematical functions used to interpolate or approximate data points smoothly and continuously. This approach is feasible because all the functions to be learned in (1) are univariate. Based on this theorem, in June 2024, Liu et al. [35] proposed the first version of a KAN architecture. But what does a KAN architecture entail? According to [36], a KAN layer with $n_{\mathrm{in}}$-dimensional inputs and $n_{\mathrm{out}}$-dimensional outputs can be represented by a matrix of one-dimensional functions as

(2) $$\Phi =\left\{\phi_{q,p}\right\},\;\;\;p=1,2,3,\cdots ,n_{\mathrm{in}},\;\;\;q=1,2,3,\cdots ,n_{\mathrm{out}},$$

where the functions $\phi_{q,p}$ are spline functions with adjustable/trainable parameters. Note that Equation (1) represents only two of these KAN layers, which can be further expanded and deepened. Thus, the KAN architecture can include $\phi_{l,j,i}\left(x_{l,i}\right)$ functions between the *i*-th neuron of the *l*-th layer and the *j*-th neuron of the (l+1)-th layer. As per [36], consequently, the activation function at the neuron *j*-th in the layer (l+1)-th can be expressed in a matrix

(3) $$x_{l+1}=\underset{\Phi_{l}}{\underset{︸}{\left(\begin{array}{cccc} \phi_{l,1,1(\;\cdot \;)} & \phi_{l,1,2(\;\cdot \;)} & \cdots & \phi_{l,1,n_{l}\left(\;\cdot \;\right)}\\ \phi_{l,2,1(\;\cdot \;)} & \phi_{l,2,2(\;\cdot \;)} & \cdots & \phi_{l,2,n_{l}\left(\;\cdot \;\right)}\\ \vdots & \vdots & \ddots & \vdots \\ \phi_{l,n_{l+1},1\left(\;\cdot \;\right)} & \phi_{l,n_{l+1},2\left(\;\cdot \;\right)} & \cdots & \phi_{l,n_{l+1},n_{l}\left(\;\cdot \;\right)} \end{array}\right)}}\;\cdot \;x_{l},$$

where $n_{l}$ represents the number of neurons in layer *l*, $n_{l+1}$ denotes the number of neurons in layer l+1, and $\Phi_{l}$ is the function matrix according to the *l*-th KAN layer. Therefore, the matrix $\Phi_{l}$ has dimensions $n_{l+1}$×$n_{l}$, and the equation represents the transformation of the input data $x_{l}$ to the output $x_{l+1}$ through this function matrix. Figure 1, adapted from [36], illustrates a reduced KAN architecture. Therefore, given a input vector x, a generalized KAN model with *L* layers can be composed of a number of nodes $\left[n_{0},n_{1},\ldots ,n_{L}\right]$ in each layer, respectively, as

(4) $$\mathrm{KAN}\left(x\right)=\left(\Phi_{L}\circ \Phi_{L-1}\circ \cdots \circ \Phi_{1}\right)x.$$

## 3. Proposed System

This study utilized dissolved gas concentration data as input for the system, provided by the Brazilian power utility company TAESA. The inputs provided gas concentration measurements in ppm for seven key gases—C_2_H_4_, CH_4_, C_2_H_6_, C_2_H_2_, H_2_, CO, and CO_2_—dissolved in transformer oil. The proposed approach analyzes the fault diagnosis of oil-immersed power transformers. For this purpose, the proposed system comprises a module responsible for the fault diagnosis of the power transformer. Section 3 details the fault diagnosis module.

### Proposed Fault Diagnosis Module

**Figure 2:**
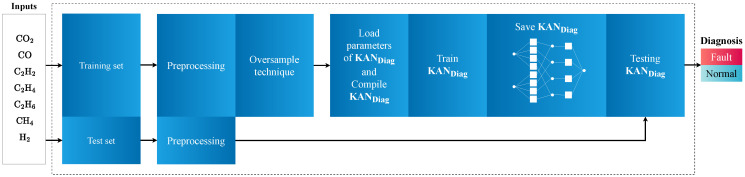
This diagram comprises a fault diagnosis module, responsible for classifying the power transformer status.

The system contains a fault diagnosis module dedicated to diagnosing faults in a power transformer immersed in oil. Initially, as per Figure 2 and the first step of Algorithm 1, the system divides the data into two sets: a training set and a test set. The gas samples (**g**_1_, **g**_2_, ⋯, and **g**_N_), obtained through DGA, are split into *q* training samples, $\{g_{1,i}$, $g_{2,i}$, $g_{3,i}$, ⋯, $g_{N,i}{\}}_{i=1}^{q}$, and *p* test samples, $\{g_{1,i}$, $g_{2,i}$, $g_{3,i}$, ⋯, $g_{N,i}{\}}_{i=1}^{p}$. This approach allows the model to learn from a portion of the data and then evaluate on different data, according to real-world scenarios. The procedure ensures that the model does not merely memorize the training samples but tests the capability of generalizing to new data, which is critical for addressing fault diagnosis challenges.

Subsequently, as illustrated in Figure 2 and the second step of Algorithm 1, the training samples undergo preprocessing. This preprocessing involves applying a logarithmic transformation and normalizing the gas samples. The logarithmic transformation reduces the variation in the data, especially when the gas values span a wide range of magnitudes. On the other hand, normalization places all samples in the same dynamic range, boosting the model’s learning, since ML models generally benefit from normalized data. The result of this step is a set of processed samples, $\{x_{1,i}$, $x_{2,i}$, $x_{3,i}$, ⋯, $x_{N,i}{\}}_{i=1}^{q}$, ready for use in the next stage.

According to Figure 2 and the third step of Algorithm 1, the SyMProD method balances the training samples. In real cases, the data available for fault diagnosis are imbalanced, meaning there might be significantly more samples of one condition (such as transformers without faults/normal) than of another (such as faults). SyMProd creates a new set of balanced β samples, ${\left\{x_{1,i},x_{2,i},x_{3,i},\cdots ,x_{N,i}\right\}}_{i=1}^{\beta }$, which ensures the model encounters an equal amount of different types of samples during training. This procedure is essential to prevent the model from becoming biased toward the more common classes, which could lead to poor performance in diagnosing less frequent faults.

As shown in Figure 2 and following the fourth step of Algorithm 1, the system trains the KAN_Diag_ architecture specifically for the fault diagnosis problem in transformers. Initially, the algorithm uses the parameters number of gases (*N*), number of training epochs (ψ), number of hidden neurons (η), number of ϕ functions ($\eta_{\Phi }$), and optimizer (Opt.) to compile and then train the model. The training process optimizes the model to enable accurate prediction. This adjustment occurs through iterations, or epochs, during which the model refines its predictive capability. After training, the system saves the model for use in the testing phase.

Next, as illustrated in Figure 2 and following the fifth step of Algorithm 1, the system applies preprocessing to the test samples. It is crucial to apply the same transformations to the test set as those used for the training set, to ensure that the data have the same scale and distribution. The test samples undergo logarithmic transformation and normalization, ensuring the model could accurately evaluate the data. The system then uses the transformed test samples $\{x_{1,i}$, $x_{2,i}$, $x_{3,i}$, ⋯, $x_{N,i}{\}}_{i=1}^{p}$ to assess the KAN_Diag_ model.

Finally, through the sixth step of Algorithm 1, the system tests the KAN_Diag_ model with the test set. The goal is to assess the model’s performance on new data, i.e., samples it has not encountered during training. By applying the model to the test set, one can evaluate its robustness and generalization capability. The outcome of this test is the fault diagnosis of transformers, indicating whether the model has correctly identified the anomalous or normal status of the equipment based on the monitored gases. This diagnosis informs decisions regarding the maintenance and safe operation of the transformers.

## 4. Experimental Results and Discussions

**Figure 3:**
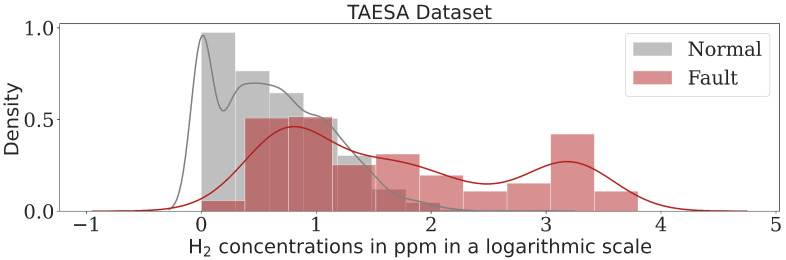
Concentrations for healthy and fault classes in the TAESA dataset.

This research utilized data on dissolved gas concentrations in transformer oil obtained from TAESA, a power utility company from Brazil. The dataset covered power transformers measurements from various manufacturers, operating at different voltage levels across 44 substations. The data were collected over the period from 2017 to 2023. They include a total of 4648 samples, with 4285 representing healthy transformers and 363 indicating faults. This is a typical scenario of data imbalance, commonly encountered in practice, where the normal condition represents the majority class. This extensive dataset captures the concentrations in ppm of seven distinct gases—C_2_H_4_, CH_4_, C_2_H_6_, C_2_H_2_, H_2_, CO, and CO_2_—found in transformer oil. According to [37], DGA data often show highly skewed distributions. As observed in Figure 3, the histograms for normal and fault states reveal different distributions, which can create challenges for model generalization during training. To address this, a logarithmic transformation was applied to the dataset due to its distribution characteristics. Furthermore, even though some fault samples are distant from the main data clusters, they should not be considered noise. Therefore, data normalization was also implemented. These preprocessing steps—logarithmic transformation and normalization—are referred to as the preprocessing. It is relevant to mention that samples in which the state was on alert but no failure had occurred were classified as normal states. Conversely, in critical situations where faults appeared, the samples were considered faults. This situation was due to the labels used in the ML model training. We trained the models following a binary approach, with labels indicating either no-fault (normal state) or fault.

**Algorithm 1:** Approach for the fault diagnosis module with the proposed KAN_Diag_
**Input:** Amount of gases involved in monitoring via DGA (*N*), complete data ($g_{1}$, $g_{2}$, ⋯, $g_{N}$), number of training epochs (ψ), number of hidden neurons (η), number of Φ functions ($\eta_{\Phi }$), and optimizer (Opt.)**Output:** Fault Diagnosis.1:first step:Split the samples of data ($g_{1}$, $g_{2}$, ⋯, $g_{N}$) into a set of *q* training samples: $\{g_{1,i}$, $g_{2,i}$, $g_{3,i}$, ⋯, $g_{N,i}{\}}_{i=1}^{q}$, and a set of *p* test samples: $\{g_{1,i}$, $g_{2,i}$, $g_{3,i}$, ⋯, $g_{N,i}{\}}_{i=1}^{p}$.2:second step:Apply preprocessing into training samples: Employ logarithm transformation and normalization at $\{g_{1,i}$, $g_{2,i}$, $g_{3,i}$, ⋯, $g_{N,i}{\}}_{i=1}^{q}$ to generate the treated training samples. Collect the set of transformed data: $\{x_{1,i}$, $x_{2,i}$, $x_{3,i}$, ⋯, $x_{N,i}{\}}_{i=1}^{q}$,3:third step:Apply SyMProd: Use SyMProd to balance the *q* samples into β balanced samples for the new training set: $\{x_{1,i}$, $x_{2,i}$, $x_{3,i}$, ⋯, $x_{N,i}{\}}_{i=1}^{\beta }$.4:fourth step:Train the KAN_Diag_ architecture: Load the parameters *N*, η, $\eta_{\Phi }$, $\lambda_{\Phi }$, and Opt., for the KAN_Diag_. Compile KAN_Diag_ model with the loaded parameters. Train the KAN_Diag_ with the training set $\{x_{1,i}$, $x_{2,i}$, $x_{3,i}$, ⋯, $x_{N,i}{\}}_{i=1}^{\beta }$. Save KAN_Diag_ model.5:fifth step:Apply preprocessing into test set: Employ logarithm transformation and normalization at $\{g_{1,i}$, $g_{2,i}$, $g_{3,i}$, ⋯, $g_{N,i}{\}}_{i=1}^{p}$ to generate the treated testing samples. Collect the set of transformed data: $\{x_{1,i}$, $x_{2,i}$, $x_{3,i}$, ⋯, $x_{N,i}{\}}_{i=1}^{p}$,6:sixth step:Testing the KAN_Diag_ model: Test the KAN_Diag_ using the test set $\{x_{1,i}$, $x_{2,i}$, $x_{3,i}$, ⋯, $x_{N,i}{\}}_{i=1}^{p}$.**return** Fault Diagnosis.


To demonstrate the robustness of the approach, it was crucial to employ multiple performance metrics for evaluation. This work evaluated the performance using Hamming loss, accuracy, and F_1_-Score, which are well-established in the literature. Each metric provides a distinct viewpoint on the performance of ML models, enabling a thorough evaluation.

As per [38,39,40], Hamming loss is a metric used to assess the performance of a classification model, particularly in multi-label classification problems where each instance may have more than one label associated with it. Hamming loss measures the proportion of incorrectly predicted labels relative to the total number of labels, providing insight into how well a model predicts the expected labels. The possible values of Hamming loss range between 0 and 1, with lower values indicating that the predicted labels are closer to being correct. A value of 0 indicates that all predictions are correct (i.e., no errors occurred), while a value of 1 signifies that all predictions are incorrect (i.e., every label is wrong). Hamming loss can be expressed as

(5) $$\mathrm{Hamming}\;\mathrm{Loss}=\frac{1}{n_{\mathrm{samples}}\times n_{\mathrm{labels}}}\sum_{i=1}^{n_{\mathrm{samples}}}\sum_{j=1}^{n_{\mathrm{labels}}}1\left(y_{ij}\neq {\hat{y}}_{ij}\right),$$

where $n_{\mathrm{samples}}$ is the number of samples, $n_{\mathrm{labels}}$ is the number of labels per sample, $y_{ij}$ is the true label for sample *i* and label *j*, ${\hat{y}}_{ij}$ is the predicted label for sample *i* and label *j*, and $1(y_{ij}\neq {\hat{y}}_{ij})$ is the indicator function that equals 1 if $y_{ij}$ is different from ${\hat{y}}_{ij}$, and 0 otherwise.

References [41,42,43] highlighted that accuracy is crucial for analyzing overall performance. In this manuscript, the accuracy evaluated the model’s overall success rate. This metric can be described as

(6) $$\mathrm{Accuracy}=\frac{\mathrm{TP}+\mathrm{TN}}{\mathrm{TP}+\mathrm{FP}+\mathrm{TN}+\mathrm{FN}},$$

where TP, TN, FP, and FN represent the counts for true positives, true negatives, false positives, and false negatives, respectively.

The F_1_-score offers a nuanced assessment of model performance. In practice, data tend to be imbalanced, as normal conditions are more frequent than fault conditions. Consequently, the normal state becomes the majority class in datasets collected from the real operation of equipment. As the F_1_-score is an evaluation metric that allows us to account for this effect, it ensures a fair assessment in such situations [44]. For this purpose, we selected the weighted average F_1_-score (F_1_) as one of our evaluation metrics. As outlined in [44,45,46], let *d* represent the number of instances in a specific class and *D* represent the total number of instances in the dataset. The F_1_ is then defined as

(7) $$F_{1}=\frac{1}{D}\sum d\times \left[F_{1}-\mathrm{Score}\right]=\frac{1}{D}\sum d\times \left[\frac{2\times \mathrm{TP}}{2\times \mathrm{TP}+1\times (\mathrm{FN}+\mathrm{FP})}\right]$$

### 4.1. Results for Fault Diagnosis Module

**Figure 4:**
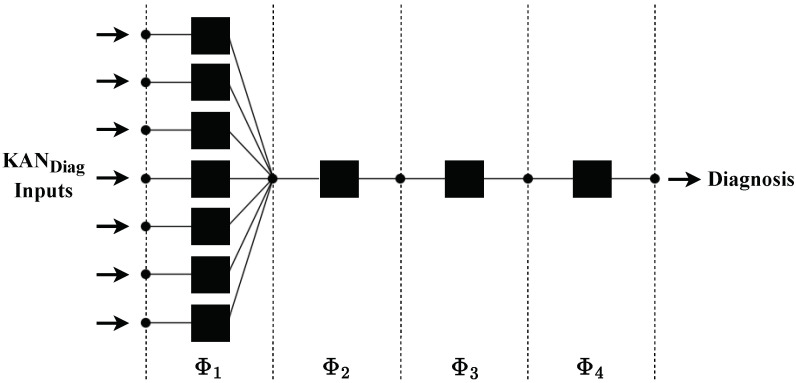
Diagram of the compiled structure of the proposed KAN_Diag_ for the fault diagnosis.

The dataset consisted of 4648 DGA samples obtained by TAESA. Half of the samples (50%) were randomly selected for the training set, while the other half (50%) were reserved for the testing set. With a 50%/50% split, the test set was more extensive, which allowed verifying the model’s generalization capability with a more ample test set. If the model’s performance was not high for the metrics employed, we might suspect it was overfitting or underfitting. In this case, it would be necessary to readjust these proportions to mitigate such issues or adopt specific techniques like cross-validation. However, we had an extensive database; thus, the training data were always sufficiently comprehensive for model training, allowing us to adopt the applied partition. As illustrated in Figure 4 and detailed in Algorithm 1, the system processed both sets using the preprocessing method. Subsequently, the system applied the SyMProd oversampling technique to the training set. In addition, the fault diagnostic module utilized KAN_Diag_, which has architectural specificities, hyperparameters such as the optimizer (Opt.), number of training epochs (ψ), number of hidden neurons (η), and number of Φ functions ($\eta_{\Phi }$). According to the recommendation of reference [35], this research used the limited-memory Broyden–Fletcher–Goldfarb–Shanno (LBFGS) for the Opt. Since the input data came from the concentrations of seven gases, the input layer of the KAN_Diag_ contained seven neurons. Additionally, the training process for the module involved an exhaustive exploration of other hyperparameter configurations, such as the ψ, η, and $\eta_{\Phi }$. Here, we tested different options in Algorithm 1 for ψ, η, and $\eta_{\Phi }$. During the experiments, these hyperparameters were systematically added to evaluate their influence on the model’s performance. We varied ψ from 1 to 50, determining the suitable value of ψ=20. Both η and $\eta_{\Phi }$ were varied from 1 to 5, resulting in η=1 and $\eta_{\Phi }=4$. Figure 4 illustrates the compiled structure of the proposed KAN_Diag_.

To assess the performance advantages of KAN_Diag_ regarding the interpretation methods, we compared it with the ASTM standard, IEC standard, CEGB standard, and Rogers ratio, as listed in Table 2. These benchmarks were implemented based on references [7,8], and references [7,9], respectively. The choice of these interpretation approaches is justified because, in addition to diagnosing faults, they also diagnose normal status. All results obtained from the mentioned techniques, documented in Table 2, used the TAESA dataset. In terms of Hamming loss, KAN_Diag_ reduced the classification error by 17.53 percentage points (pps) compared to the ASTM standard, 10.61 pps compared to the IEC standard, 6.75 pps compared to the CEGB standard, and 3.20 pps compared to the Rogers ratio, demonstrating a more efficient performance in minimizing errors. For the F_1_ metric, KAN_Diag_ showed advantages of 20.70 pps over the ASTM standard, 16.91 pps over the IEC standard, 8.88 pps over the CEGB standard, and 6.38 pps over the Rogers ratio, highlighting the KAN_Diag_ as a more effective model for fault detection in imbalanced datasets. Finally, in terms of accuracy, it outperformed the ASTM standard by 17.53 pps, the IEC standard by 8.69 pps, the CEGB standard by 6.75 pps, and the Rogers ratio by 3.19 pps, establishing KAN_Diag_ as the most robust model for diagnostics. These results confirmed that KAN_Diag_ offered a notable advantage, improving the reliability and robustness compared to traditional models. Examining Table 2, it is evident that KAN_Diag_ not only achieved the lowest Hamming loss of 0.0323 but also excelled with the highest accuracy and F_1_ scores, reaching 96.7728% and 96.8455%, respectively.

To evaluate the performance benefits of KAN_Diag_ in comparison to ML models, we conducted a comparison with DT [12], *k*-NN [14], and ELM [16,47] models, as shown in Table 3. These ML models are widely employed in various diagnostic applications. According to results from references [21,22,26], the choice of these models was justified by their established performance and robustness in identifying faults. Here, it was necessary to define the hyperparameters of each model to implement them in the proposed system. These hyperparameters included the maximum depth of the DT, the number of *k* neighbors for *k*-NN, and the number of neurons for the hidden layer of ELM. We employed grid search (GS) with K-fold cross-validation (K-CV) to determine these hyperparameters, as referenced in [48]. As recommended in [49], we used K = 10 folds for the GS with K-CV. For the DT model, we explored tree depths ranging from 1 to 100 in increments of 1, ultimately identifying a depth of 3 as the most suitable configuration. For the *k*-NN model, we tested the number of neighbors (*k*) over a range from 1 to 100, with increments of 1, ultimately determining that 46 neighbors provided the optimal performance. For the ELM model, we varied the number of neurons from 100 to 1000, with increments of 100, and identified that 100 neurons yielded the best performance. These parameter variation ranges were selected to ensure computational efficiency and performance reliability while the system performed hyperparameter tuning for DT, *k*-NN, and ELM. All results presented in Table 3 utilized the TAESA dataset. In terms of Hamming loss, KAN_Diag_ reduced the classification error by 3.09 pps compared to ELM, 1.42 pps compared to DT, and 1.37 pps compared to *k*-NN, highlighting a more efficient performance in error minimization. For the F_1_, KAN_Diag_ offered advantages of 2.96 pps over ELM, 2.01 pps over DT, and 1.81 pps over *k*-NN, indicating a more effective fault detection model in imbalanced dataset scenarios. Finally, in terms of accuracy, KAN_Diag_ surpassed ELM by 3.10 pps, DT by 1.42 pps, and *k*-NN by 1.38 pps, establishing itself as the most robust model for diagnostics. These results confirm that KAN_Diag_ offers a notable advantage in reliability and robustness compared to other methods like ELM, DT, and *k*-NN. According to Table 3, when comparing KAN_Diag_ with the ML models, KAN_Diag_ stands out with the lowest Hamming loss of 0.0323, the highest F_1_ at 96.8455%, and the highest accuracy at 96.7728%.

According to Table 4, the KAN_Diag_ model demonstrated a relevant advantage in training speed compared to the other techniques. Specifically, KAN_Diag_ completed its analysis in about 15.38 s, which was notably faster than *k*-NN at 15.92 s and DT at 17.43 s. The KAN_Diag_ saved 0.55 s compared to *k*-NN and 2.05 s compared to DT, demonstrating a clear advantage in training time.

### 4.2. Comparison with Works Documented in the Current Literature

By incorporating the DT into the proposed system within the fault diagnosis module, according to Table 5, the DT achieved an accuracy of 95.35%, demonstrating superior performance compared to existing models in the literature. Concerning the naive Bayes (NB) model used by [22], which attained 28.15%, our model exhibited an increase of 67.20 accuracy percentage points (pps). Compared to the MLP from the same study, with an accuracy of 62.13%, the DT model showed an improvement of 33.22 pps. In fact, our DT model outperformed multiple methods: modified IECM by [19] (17.57 pps more), modified Rogers ratio from [19] (15.01 pps more), SVM from [22] (14.77 pps more), ANFIS from [20] (14.75 pps more), *k*-NN with SMOTE method from [26] (12.95 pps more), DT from [21] (12.75 pps more), *k*-NN with ADASYN method from [26] (12.05 pps more), *k*-NN from [22] (11.86 pps more), SVM from [21] (11.25 pps more), ANFIS with DS theory from [20] (10.95 pps more), *k*-NN with GAN method from [26] (10.52 pps more), *k*-NN with DCGAN from [26] (10.16 pps more), AutoWeka from [22] (9.92 pps more), *k*-NN from [21] (9.15 pps more), CNN from [3] (8.60 pps more), ensemble from [21] (7.65 pps more), optimized AutoWeka from [22] (7.01 pps more), modified triangle diagnosis from [23] (6.46 pps more), fuzzy C-means from [25] (6.45 pps more), *k*-NN with SDG method from [26] (5.09 pps more), neural pattern recognition from [27] (2.55 pps more), and SVM-BA from [28] (1.60 pps more). These results highlight the superior efficacy of our system with the DT model compared to a wide range of existing methods, reflecting reliable and robust performance for fault diagnosis.

When utilizing *k*-NN within the proposed fault diagnosis module, as per Table 5, the model demonstrated a notable superiority over competing methods, achieving an accuracy of 95.40%. Compared to the NB method presented in [22] (67.25 pps more), the MLP described in the same study (33.27 pps more), the modified IECM from [19] (17.62 pps more), the modified Rogers ratio from [19] (15.06 pps more), the k-NN model consistently demonstrated superior performance. The *k*-NN also surpassed the SVM from [22] (14.82 pps more), the ANFIS from [20] (14.80 pps more), the *k*-NN with SMOTE from [26] (12.99 pps more), and DT from [21] (12.80 pps more), the *k*-NN with ADASYN from [26] (12.10 pps more), the *k*-NN from [22] (11.91 pps more). Our *k*-NN model continued to outperform the other methods. It also surpassed the SVM from [21] (11.30 pps more), the ANFIS with DS theory from [20] (10.90 pps more). The *k*-NN further demonstrated superior accuracy compared to the *k*-NN with GAN from [26] (10.57 pps more), *k*-NN with DCGAN from [26] (10.21 pps more), and AutoWeka from [22] (10.00 pps more). Our *k*-NN also surpassed the *k*-NN from [21] (9.20 pps more), CNN from [3] (8.65 pps more), ensemble from [21] (7.70 pps more), optimized AutoWeka from [22] (7.06 pps more), modified triangle diagnosis from [23] (6.51 pps more), fuzzy C-means from [25] (6.50 pps more), *k*-NN with SDG from [26] (5.14 pps more), neural pattern recognition from [27] (2.60 pps more), and SVM-BA from [28] (1.65 pps more). These results highlight the enhanced effectiveness of our system with the *k*-NN model, showcasing its reliable and robust performance for fault diagnosis modules.

Applying the proposed KAN_Diag_ in the fault diagnosis module, according to Table 5, our system exhibited the an edge over alternative approaches, attaining the highest accuracy of 96.77%. Compared to the NB method from [22] (68.62 pps more), MLP from [22] (34.64 pps more), Modified IECM from [19] (18.99 pps more), and modified Rogers ratio from [19] (16.43 pps more), the KANDiag consistently demonstrated superior performance. The KANDiag also surpassed the SVM from [22] (16.19 pps more) and the ANFIS from [20] (16.17 pps more). The KAN_Diag_ also outperformed the *k*-NN with SMOTE from [26] (14.37 pps more) and the DT from [21] (14.17 pps more). Relative to the *k*-NN with ADASYN from [26] (13.47 pps more) and the *k*-NN from [22] (13.28 pps more), the KAN_Diag_ demonstrated consistent advantages. The KAN_Diag_ also showed superior accuracy compared to the SVM from [21] (12.67 pps more) and the ANFIS with DS theory from [20] (12.37 pps more). Furthermore, the method surpassed the *k*-NN with GAN from [26] (11.94 pps more), *k*-NN with DCGAN from [26] (11.58 pps more), and AutoWeka from [22] (11.34 pps more). The KAN_Diag_ also demonstrated superior performance compared to the *k*-NN from [21] (10.57 pps more), CNN from [3] (10.02 pps more), ensemble from [21] (9.07 pps more), opt. AutoWeka from [22] (8.43 pps more), modified triangle diagnosis from [23] (7.88 pps more), fuzzy C-means from [25] (7.87 pps more), *k*-NN with SDG from [26] (6.51 pps more), neural pattern recognition from [27] (3.97 pps more), and SVM-BA from [28] (3.02 pps more). These results underscore the advanced effectiveness of our system with the proposed KAN_Diag_, reflecting its dependability and robust performance for fault diagnosis modules.

Although the topic of DGA analysis has been extensively covered in the literature, this work focused specifically on the technique’s effectiveness in implementing diagnostic modules. Various practical details and qualitative fault analyses fall outside the scope of this paper and can be explored through the numerous references provided in Table 5.

## 5. Conclusions

In this work, we proposed a novel approach based on a KAN model for the fault diagnosis of oil-immersed power transformers in a real-world imbalanced data scenario. Currently, gaps remain in real scenarios with imbalanced datasets, such as the reliability and robustness of the fault diagnosis module. To overcome this challenge, our study employed a dedicated KAN for the fault diagnosis module, the KAN_Diag_, in addition to utilizing SyMProD to balance the data. This original proposal introduced a pioneering analysis of the KAN_Diag_ architecture applied to a dataset collected under realistic conditions of imbalance from power transformers monitored by TAESA. Upon verifying the results in Table 2 and Table 3, KAN_Diag_ demonstrated superior performance in the fault diagnosis module compared to traditional interpretation methods and ML approaches. Some key results revealed that KAN_Diag_ reduced the classification error by 17.53 pps in Hamming loss compared to the ASTM standard, achieved 20.70 pps over the ASTM standard for the F_1_, and surpassed the ASTM standard by 17.53 pps for the accuracy metric. The results exhibited a trend of KAN_Diag_ outperforming the other interpretation methods, the IEC standard, the CEGB standard, and the Rogers ratio. When compared with machine learning approaches, KAN_Diag_ minimized the classification error by 3.09 pps compared to ELM, offered advantages of 2.959 pps over ELM for F_1_, and outperformed ELM by 3.0981 pps for accuracy. The results display a trend where KAN_Diag_ also outperformed the other ML models in this study, the DT and the *k*-NN.

Another notable finding pertains to a direct competitor, the CNN from reference [3], where our KAN_Diag_ achieved a 10.01 pps accuracy advantage, beating the CNN. Our findings demonstrate the superiority of the proposed system regarding reliability, with lowest error in classification—analyzed through Hamming loss—the highest robustness in a real scenario with an imbalanced dataset—explored using F_1_—and the highest overall robustness of the models—verified via accuracy. All findings in this manuscript demonstrate the proposed system’s efficiency and robustness. In future work, it would be possible to expand the analyses to include predictive diagnostics for different types of transformer damage, by considering practical hardware implementations for use in operational diagnostic systems via KAN, and by assessing the condition of power transformers.

## Figures and Tables

**Table 1:** List of state-of-the-art approaches and their key characteristics.

Works	Input Variable for Fault Diagnosis Module	Oversampler	Decision Maker	Robustness
Ref. [19]	Five key gases ^1^	None	Modified IECM and Modified Rogers Ratio	Intermediate
Ref. [20]	Seven key gases ^3^	None	ANFIS and ANFIS with Dempster-Shafer theory	Intermediate
Ref. [3]	Seven key gases ^3^	SMOTE	CNN	Intermediate
Ref. [21]	Five key gases ^2^	None	DT; SVM; *k*-NN, Ensemble	Intermediate
Ref. [22]	Not detailed	None	*k*-NN, AutoWeka, Naive Beyes, MLP, SVM	Intermediate
Ref. [23]	Five key gases ^1^	None	Modified triangle diagnosis	Intermediate
Ref. [25]	Five key gases ^1^	None	Fuzzy C-Means	Intermediate
Ref. [26]	Five key gases ^1^	SMOTE, ADASYN, GAN, and DCGAN	*k*-NN, SVM, and RF	High
Ref. [27]	Five key gases ^1^	None	Neural pattern recognition	High
Ref. [28]	Five key gases ^1^	None	SVM-BA	High
Our System	Seven key gases ^3^	SyMProD	KAN_Diag_	Superior

NOTE: Five key gases ^1^: C_2_H_4_, CH_4_, C_2_H_6_, C_2_H_2_, and H_2_; five key gases ^2^: C_2_H_4_, CH_4_, C_2_H_6_, H_2_, and CO; seven key gases ^3^: C_2_H_4_, CH_4_, C_2_H_6_, C_2_H_2_, H_2_, CO, and CO_2_.

**Table 2:** Comparison between KAN_Diag_ and interpretation approaches using the TAESA dataset in the fault diagnosis module.

Approach	Hamming Loss	F_1_	Accuracy
ASTM Standard	0.2076	76.1449%	79.2419%
IEC Standard	0.1384	79.9385%	88.0807%
CEGB Standard	0.0998	87.9627%	90.0239%
Rogers Ratio	0.0643	90.4666%	93.5732%
KAN_Diag_	0.0323	96.8455%	96.7728%

**Table 3:** Comparison between KAN_Diag_ and ML approaches using the TAESA dataset in the fault diagnosis module.

Approach	Hamming Loss	F_1_	Accuracy
ELM	0.0632	93.8865%	93.6747%
DT	0.0465	94.8331%	95.3528%
*k*-NN	0.0460	95.0317%	95.3959%
KAN_Diag_	0.0323	96.8455%	96.7728%

**Table 4:** Training time comparison between KAN_Diag_ and ML approaches using the TAESA dataset in the fault diagnosis module.

ELM	KAN_Diag_	k−NN	DT
0.1987 s	15.3761 s	15.9229 s	17.4262 s

**Table 5:** Comparison with other approaches from the literature.

Works	Samples	Model for Fault Diagnosis Module	Accuracy of Fault Diagnosis Module
Ref. [22]	4580	NB	28.15%
Ref. [22]	4580	MLP	62.13%
Ref. [19]	481	Modified IECM	77.78%
Ref. [19]	481	Modified Rogers Ratio	80.34%
Ref. [22]	4580	SVM	80.58%
Ref. [20]	697	ANFIS	80.60%
Ref. [26]	1614	*k*-NN–SMOTE	82.40%
Ref. [21]	138	DT	82.60%
Ref. [26]	1614	*k*-NN–ADASYN	83.30%
Ref. [22]	4580	*k*-NN	83.49%
Ref. [21]	138	SVM	84.10%
Ref. [20]	697	ANFIS with DS theory	84.40%
Ref. [26]	1614	*k*-NN–GAN	84.83%
Ref. [26]	1614	*k*-NN–DCGAN	85.19%
Ref. [22]	4580	AutoWeka	85.43%
Ref. [21]	138	*k*-NN	86.20%
Ref. [3]	1083	CNN	86.75%
Ref. [21]	138	Ensemble	87.70%
Ref. [22]	4580	opt. AutoWeka	88.34%
Ref. [23]	383	Modified triangle diagnosis	88.89%
Ref. [25]	177	Fuzzy C-Means	88.90%
Ref. [26]	1614	*k*-NN–SDG	90.26%
Ref. [27]	446	Neural pattern recognition	92.80%
Ref. [28]	481	SVM-BA	93.75%
Our System	4648	DT	95.35%
Our System	4648	*k*-NN	95.40%
Our System	4648	KAN_Diag_	96.77%

## Data Availability

Access to the data underlying the findings of this study is not available due to privacy considerations and in accordance with company operational policies.

## Abbreviations

AI	Artificial Intelligence
ANN	Artificial Neural Network
ANFIS	Adaptive Neuro-Fuzzy Inference System
ASTM	American Society for Testing and Materials
CEGB	Central Electricity Generating Board
CNN	Convolutional Neural Network
DGA	Dissolved Gas Analysis
DCGAN	Deep Convolutional Generative Adversarial Network
DP	Degree of Polymerization
DS	Dempster–-Shafer
DT	Decision Tree
ELM	Extreme Learning Machine
GAN	Generative Adversarial Network
IECM	Integrated Energy Communities Management
IEC	International Electrotechnical Commission
KAN	Kolmogorov–Arnold Network
KAN_Diag_	Kolmogorov–Arnold Network for fault diagnosis module
MLP	Multilayer Perceptron
RF	Random Forest
SVM	Support Vector Machine
SVM-BA	Support Vector Machine with Bat Algorithm
*k*-NN	*k*-Nearest Neighbor
SMOTE	Synthetic Minority Over-sampling Technique
SyMProD	Synthetic Minority Proportional Dissimilarity
TAESA	Transmissora Aliança de Energia Elétrica S.A.
